# The Effects of Meperidine Analgesia during Labor on Fetal Heart Rate

**Published:** 2009-03

**Authors:** Leila Sekhavat, Shecoofah Behdad

**Affiliations:** 1 *Department of obstetrics & gynecology, Shahid Sedughi hospital, Shahid Sedughi University of Medical Sciences and Health Services, yazd, Iran;*; 2 *Department of Anesthesiology, Shahid Sedughi hospital, Shahid Sedughi University of Medical Sciences and Health Services, yazd, Iran*

**Keywords:** meperidine, labor analgesia, fetal heart rate

## Abstract

To estimate the effects of intramuscular meperidine analgesia on fetal heart rate (FHR) patterns compared with placebo. In a prospective randomized study, 150 healthy women with singleton term pregnancy requesting analgesia during active labor were planned to receive either intramuscular meperidin 50 mg (meperidin group) or normal saline (control group) when they requested analgesia. Fetal heart rate patterns occurring within 40 minutes of initiation of labor analgesia were retrospectively read by maternal fetal medicine specialist who was blind to type of labor analgesia. Meperidine, compared with placebo, was associated with statistically significantly less beat to beat variability (absent or less than 5 beats per minute) (28% versus 5% of fetuses, *P*<0.05), lower proportion of accelerations (37.3% versus 17.3% *P*<0.05) and of the FHR. Also FHR deceleration was significantly more than control group (25.5% versus 4%, *P*<0.05). Meperidine has deleterious effects on FHR.

## INTRODUCTION

Systemic opioids are widely used to relieve pain during labor. They are simple to use and readily available. Of available opioids, the most commonly used is pethidine, usually given intramuscularly in doses of 50–100 mg (1). In a recent survey in the United States, the use of parental opioids was found to be between 39% and 56% in various hospital obstetrics units (2). Systemic opioids are associated with adverse effects on both mother and baby. For the mother, these include dysphoria, sedation, respiratory depression, nausea and vomiting and delayed gastric emptying (3). Pethidine readily crosses the placenta and may accumulate in the fetal circulation, especially in the presence of fetal compromise (4, 5) Observational studies have found negative effects on fetal heart rate (6, 7), and the following negative newborn effects have been reported: low Apgar scores (8), respiratory depression and acidosis at birth (9). Newborn depression has been associated not only with the use of pethidine, but also with the maternal administration-birth period of time (1, 7). Available data on the efficacy of systemic opioids for labour analgesia are limited because of the absence of randomized controlled blinded trials comparing opioids with placebo. Ethical considerations may previously have contributed to this lack of placebo-controlled studies. Electronic fetal monitoring is one technique in the overall strategy of intrapartum fetal surveillance (10); and can improve outcomes by identifying fetuses with hypoxic acidemia at a point when the process is still completely reversible by intra-uterine resuscitation or expedited delivery (11). James recently commented that meperidine, compared with epidural analgesia, was associated with statistically significantly less beat-to-beat variability and fewer FHR accelerations (12); and Giannina reported controversy meperidine using during the first stage of labor did not lead to adverse fetal effects (13). Therefore, the present study was performed to determine, whether pethidine use during labor increased the risk of fetal heart abnormalities.

## MATERIAL AND METHODS

A randomized clinical trial study was designed and conducted on healthy parturient women with singleton, cephalic-presenting fetus at term pregnancy and in spontaneous labor, in active phase of labor with a cervical dilation of 3 cm or more, requesting analgesia during active labor. All patients were referred to Shahid Sadoughi hospital in Yazd, from October 2004 to September 2005. The adopted protocol was approved by the hospital research and ethics committee. All women were interviewed individually by the researcher. Written informed consent was obtained from all the patients. 150 women enrolled the study. The women were randomly (using a randomized consecutive numbered chart) allocated to either receive intramuscular meperidin 50 mg (meperidin group, N=75) or normal saline (control group, N=75) when they requested. Exclusion criteria were pethidine allergy, contraindication for vaginal delivery, use of any kind of analgesia prior to randomization, history of drug abuse, fetal death, evidence of fetal distress or antenatal diagnosis of congenital heart malformation and obstetrics complication such as antepartum hemorrhage. All pregnancies were managed by second degree resident under the direct supervision of obstetric faculty. Routine intrapartum management of all women included intravenous fluid administration and continuous electronic FHR surveillance initially for at least 30 minutes. Pelvic examinations were performed approximately every 2 hours to evaluate the progress of labor. Cervical change of less than 1.5 cm/hour coincidental with hypotonic uterine contractions, measured with intrauterine pressure transducers, was treated with Oxytocin augmentation of labor. Oxytocin was administered per written protocol, which has been described previously (14). Infuse oxytocin 2.5 units in 500 mL of dextrose (or normal saline) at 10 drops per minute. This is approximately 2.5 mIU per minute. Increase the infusion rate by 10 drops per minute every 30 minutes until a good contraction pattern is established uterine activity of 200–250 Montevideo units for 2–4 hours was considered adequate for effective labor. Women randomized to meperidine group received 50 mg meperidine with administered intramuscularly as an initial dose, additional 25mg doses of meperidine IM were given on request after 4 hour. In control group similar to meperidine group and the same time were given normal saline. During labor, left lateral displacement was maintained to avoid aortocaval compression.

The examiner resident, blind to the choice of analgesia, independently reviewed the FHR tracings recorded during the 40 minutes after initiation of injection in the two study groups. All notations on the tracings were redacted by a research physician and interpreted. All tracings were read independently at the outset by investigator. The FHR patterns were interpreted according the criteria shown in Table 1 which has been described previously (15).

**Table 1:** Criteria for Interpretation of Fetal Heart Rate

Fetal heart rate	Definition

Variability	
Absent	Undetectable
Minimal	5 BPM
Moderate	6-25 BPM
Marked	>25 BPM
Accelerations	15 BPM above baseline lasting 15 s
Decelerations	
Early	Gradual decrease and return to baseline with nadir at the same time as peak of the contraction
Late	Gradual decrease and return to baseline with nadir after the peak of the contraction
Variable	Abrupt decrease below the baseline 15 BPM lasting 15 s, and <2 minutes in duration
Prolonged	Decrease from baseline 15 BPM, lasting 2 minutes

BPM, Beat per minute.

All analyses of significance were performed with two-tailed tests. Data were analyzed with SPSS software (Statistical Package for Social Science) ver 15.0. Statistical significance (P<0.05) was determined with unpaired student t-test and Pearson chi-square test, as indicated. P<0.05 was assumed significant.

## RESULT

Table 2 showed baseline demographic and outcome data of the two study groups. There were no significant differences between to study groups in baseline demographic variables. The cervical dilatation at analgesia was not significantly different between the two groups. All fetuses were normal and had a reactive FHR tracing prior to enrollment in the study. Twelve women need to second dose of meperidine due to inadequate pain relief. All the 150 women spontaneously delivered normal fetuses. The Apgar score at 5 minutes after delivery was 10 in 51 cases in meperidine group and 50 in control group and 8 in 14 cases in meperidine group and 15 in control group. Sufficient tracing for analysis was obtained in all cases.

**Table 2:** Baseline characteristics of the study groups

Characteristics	Mepridin (n = 75)	Control (n = 75)	p value

Age (years)^a^	35.1 ± 3.7	34.7 ± 3.9	0.4
Gestational age (weeks)^a^	39.2 ± 1.5	39.4 ± 1.1	0.7
Cervical dilatation (cm)^a^	3.7 ± 3.4	4.1 ± 3.7	0.2
Parity^b^			
Nullipara	35 (46.7)	31 (41.3)	0.09
Multipara	40 (53.3)	44 (58.7)	0.09
Oxytocin augmentation^b^	52 (69.3)	49 (65.3)	0.6
Birth weight (g)^a^	3080 ± 475	3101 ± 400	0.7
Apgar (first 10 min)^a^	7.9 ± 2.0	7.4 ± 2.5	0.5

a mean ± SD;

b number (%).

The FHR patterns observed in the two study groups are shown in Table 3. There were a lower proportion of accelerations compared with control group, (53.3% versus 62.7% P<0.05) and a greater incidence of abnormal variability (absent or less than 5 beats per minute) in the meperidine group, (20% versus 5.4%, P<0.05). FHR deceleration was recorded in 37 cases (Early deceleration in 24, late in 10, variable in 1 and prolonged 2 cases) and compared with control group it was significant (49.3% versus 22.7%, P<0.05). There were no significant adverse effects in the newborn infants and there were no increased using naloxone. There was no significant different between two groups in length of labor.

**Table 3:** Fetal heart rate patterns observed within 30 min after injection meperidine and placebo

Characteristics	Mepridin (n = 75)	Control (n = 75)	p value

Variability^a^			
Absent	5 (6.7)	2 (2.7)	0.00
≤5 BPM	10 (13.3)	2 (2.7)	0.00
6-25 BPM	51 (68)	19 (25.3)	0.00
>25 BPM	9 (12)	52 (69.3)	0.00
Accelerations^a^	40 (53.3)	62 (82.7)	0.00
Decelerations^a^			
None	38 (50.7)	58 (77.3)	0.01
Early	24 (32)	11 (14.7)	0.01
Late	10 (13.3)	4 (5.4)	0.00
Prolonged	2 (2.7)	1 (1.3)	0.04
Variable	1 (1.3)	1 (1.3)	0.8

a Number (%).

## DISCUSSION

Numerous studies have attempted to quantify the effect of meperidine on the mother and fetus. In the present study meperidine was administered interamuscular in first stage of labor. The purpose of this study was to evaluate primarily the effect of meperidine on fetal heart rate during labor.

There are conflicting data regarding the effect of meperidine on the different FHR indices. Our study showed that intramuscular meperidine 50 mg had a comparatively greater incidence of abnormal FHR. Meperidine was associated with significantly less beat-to-beat variability, defined as absent or less than 5 beats per minute variability and fewer accelerations when compared with control group. These changes were shown in Solt study, but they use meperidine plus promethazine in their study (7). It is unclear whether the effect can be attributed to one of the two drugs or the combination. In our study the incidence of decelerations was greater in meperidine group compared with control group, and this was true for all types of decelerations; but this was not accepted by Giannina *et al.* they showed intheir study that meperidine had no significant effect on any FHR characteristic (13). In James study the result s were like our study but they used epidural instead placebo and continued intravenous meperidin (12); but in this study like Isenor *et al* we used intra muscular separated doses of meperidine (16).

First 10 minute APGAR in newborn were not different in two groups; Michelle *et al* also maintain “that meperidine has no effect on APGAR (17).

## CONCLUSION

Objective analysis of FHR tracings indicates that maternal administration of meperidine has a significant effect on FHR indices in the active phase of labor.
